# Real-world evidence in achondroplasia: considerations for a standardized data set

**DOI:** 10.1186/s13023-023-02755-w

**Published:** 2023-06-26

**Authors:** Yasemin Alanay, Klaus Mohnike, Ola Nilsson, Inês Alves, Moeenaldeen AlSayed, Natasha M. Appelman-Dijkstra, Genevieve Baujat, Tawfeg Ben-Omran, Sandra Breyer, Valerie Cormier-Daire, Pernille Axél Gregersen, Encarna Guillén-Navarro, Wolfgang Högler, Mohamad Maghnie, Swati Mukherjee, Shelda Cohen, Jeanne Pimenta, Angelo Selicorni, J. Oliver Semler, Sabine Sigaudy, Dmitry Popkov, Ian Sabir, Susana Noval, Marco Sessa, Melita Irving

**Affiliations:** 1 Pediatric Genetics, Department of Pediatrics, School of Medicine, Acibadem Mehmet Ali Aydinlar University, Kayisdagi Cad. No:32, Atasehir, 34684 Istanbul, Turkey; 2 Department of Pediatrics, Otto-von-Guericke-University, Magdeburg, Germany; 3 Division of Pediatric Endocrinology and Center for Molecular Medicine, Department of Women’s and Children’s Health, Karolinska Institute and University Hospital, Stockholm, Sweden; 4 Department of Medical Sciences, Örebro University, Örebro, Sweden; 5 Department of Pediatrics, Örebro University Hospital, Örebro, Sweden; 6 ANDO Portugal, Évora, Portugal; 7 Department of Medical Genomics, Center for Genomic Medicine, King Faisal Specialist Hospital and Research Center, Riyadh, Saudi Arabia; 8 College of Medicine, Alfaisal University, Riyadh, Saudi Arabia; 9 Department of Internal Medicine, Division Endocrinology and Center for Bone Quality, Leiden University Medical Center, Leiden, The Netherlands; 10 Hôpital Necker Enfants Malades AP-HP, Paris, France; 11 Genetic and Genomic Medicine Division, Sidra Medicine and Hamad Medical Corporation, Doha, Qatar; 12 Department of Paediatrics, UKE Hamburg-Eppendorf, Hamburg, Germany; 13 Reference Center for Skeletal Dysplasia, Imagine Institute, Paris Cité University, Paris, France; 14 Department of Clinical Genetics and Centre for Rare Diseases, Aarhus University Hospital, Aarhus, Denmark; 15 Medical Genetics Section, Department of Paediatrics, Virgen de la Arrixaca University Clinical Hospital, IMIB-Arrixaca, Faculty of Medicine, University of Murcia (UMU), Murcia, Spain; 16 Department of Paediatrics and Adolescent Medicine, Johannes Kepler University Linz, Linz, Austria; 17 Department of Paediatrics, IRCCS Istituto Giannna Gaslini, Genoa, Italy; 18 Department of Neuroscience, Rehabilitation, Ophthalmology Genetics, Maternal and Child-Health, University of Genova, Genoa, Italy; 19 BioMarin (UK) Limited, London, UK; 20 Pediatric Unit ASST Lariana, Mariani Center for Fragile Child, Como, Italy; 21 Faculty of Medicine, University of Cologne, Cologne, Germany; 22 Department of Pediatrics, University Hospital Cologne, Cologne, Germany; 23 Département de Génétique Médicale, Hôpital Timone Enfant, Marseille, France; 24 National Ilizarov Research Center for Traumatology and Orthopaedics, Kurgan, Russia; 25 Fundación ALPE Acondroplasia, Asturias, Spain; 26 Associazione per I’Informazione e lo Studio dell’Acondroplasia (AISAC), Milan, Italy; 27 Guy’s and St. Thomas’ NHS Foundation Trust, Evelina Children’s Hospital, London, UK

**Keywords:** Achondroplasia, Registry, Real-world data, Real-world evidence, Growth, Quality of life, Registry, Rare disease

## Abstract

**Background:**

Collection of real-world evidence (RWE) is important in achondroplasia. Development of a prospective, shared, international resource that follows the principles of findability, accessibility, interoperability, and reuse of digital assets, and that captures long-term, high-quality data, would improve understanding of the natural history of achondroplasia, quality of life, and related outcomes.

**Methods:**

The Europe, Middle East, and Africa (EMEA) Achondroplasia Steering Committee comprises a multidisciplinary team of 17 clinical experts and 3 advocacy organization representatives. The committee undertook an exercise to identify essential data elements for a standardized prospective registry to study the natural history of achondroplasia and related outcomes.

**Results:**

A range of RWE on achondroplasia is being collected at EMEA centres. Whereas commonalities exist, the data elements, methods used to collect and store them, and frequency of collection vary. The topics considered most important for collection were auxological measures, sleep studies, quality of life, and neurological manifestations. Data considered essential for a prospective registry were grouped into six categories: demographics; diagnosis and patient measurements; medical issues; investigations and surgical events; medications; and outcomes possibly associated with achondroplasia treatments.

**Conclusions:**

Long-term, high-quality data are needed for this rare, multifaceted condition. Establishing registries that collect predefined data elements across age spans will provide contemporaneous prospective and longitudinal information and will be useful to improve clinical decision-making and management. It should be feasible to collect a minimum dataset with the flexibility to include country-specific criteria and pool data across countries to examine clinical outcomes associated with achondroplasia and different therapeutic approaches.

**Supplementary Information:**

The online version contains supplementary material available at 10.1186/s13023-023-02755-w.

## Introduction

Achondroplasia is the most common form of short-stature skeletal dysplasia [1, 2], with an estimated worldwide birth prevalence of 4.6 per 100,000 [3]. Achondroplasia is caused by one of two known pathogenic *FGFR3* variants (c.1138G > A, c.1138G > C) that substitute glycine for arginine at amino acid position 380, disrupting the tyrosine kinase domain and constitutively upregulating the receptor. The *FGFR3* variant arises de novo in 76 − 80% of individuals [2, 4]. Over-expression of *FGFR3* affects endochondral bone formation, predominantly at the growth plates, leading to a distinctive pattern of skeletal features [1, 2] characterized primarily by disproportionate short stature and macrocephaly [1, 5, 6]. These abnormalities in bone growth and development are associated with a range of orthopaedic, respiratory, neurological, dental, and ear, nose, and throat (ENT) manifestations, as well as psychosocial issues [5, 7, 8]. Optimal care and follow-up require a coordinated multidisciplinary approach, with early monitoring and medical and surgical interventions to reduce morbidity and mortality [1, 2].

Real-world evidence (RWE)—the analysis of real-world data generated and captured during routine clinical practice—is a key instrument to better understand rare diseases, including their prevalence, natural history, and unmet medical needs [9]. RWE is playing an increasing role in clinical decision-making and implementing the right patient-care model, is being used to monitor safety and adverse events [10] of new therapeutic options, and is starting to be used in regulatory decisions [11]. Of 793 rare disease registries identified across 36 countries in the Orphanet Network, none is currently specific to achondroplasia. Elsewhere, the Registry of Achondroplasia (ReACH) is collecting data from patients in the Czech Republic to facilitate potential access to newly developed treatments. A few clinical cohorts have published data [12–14], the largest being the Achondroplasia Natural History Study (CLARITY), a retrospective cohort of 1374 patients in the United States [4, 15]. Whereas these and other individual sources of RWE on achondroplasia exist, their datasets are difficult to pool due to, for example, differences in data standards and items captured. The development of internationally applicable, standardized, prospective registries that capture long-term data in a consistent manner, particularly in the context of new therapeutic approaches, would improve understanding of the natural history of achondroplasia, quality of life in people with the condition, the outcomes associated with clinical management, and knowledge gaps. The aim of this report is to provide an expert opinion on which data elements are considered essential to capture the medical issues, clinical outcomes, quality of life, and functional impact among individuals with achondroplasia in a real-world clinical setting.

## Methods

The Europe, Middle East, and Africa (EMEA) Achondroplasia Steering Committee comprises a multidisciplinary group of healthcare practitioners (HCP) (endocrinologists, geneticists, orthopaedic surgeons, and paediatricians) with expertise in achondroplasia practising in 11 countries in EMEA, and advocacy organization representatives (AOR) from EMEA (Additional file 1: Table 1). The Steering Committee meeting was held with the following objectives in mind:

- To assess which parameters of RWE on achondroplasia are currently being collected across the EMEA region.
- To identify parameters considered essential for a comprehensive resource covering the natural history of achondroplasia and outcomes associated with treatment that will be relevant to decision-making for achondroplasia management.
- To recommend a pragmatic set of essential parameters, given the issues and practicalities of long-term, voluntary data collection in everyday clinical practice.
- To emphasize the need for AOR input in the generation of RWE.

**Table 1:** Onset of manifestations associated with achondroplasia over the life course [1, 2, 7, 12, 16–20]

Body system	0–2 years	3–12 years	13–18 years	> 18 years
Musculoskeletal and central nervous system	Foramen magnum stenosis Cervical medullary compression Kyphosis	*(Risk is higher in the earlier years)* Foramen magnum stenosis Hydrocephalus Cervical medullary compression		
		Cervical medullary compression Symptomatic spinal stenosis Lumbar hyperlordosis Kyphosis Hip flexion contractures Limited elbow extension Genu varum Tortional deformity of lower limb segments Early onset arthritis-related pain syndrome		
	Hydrocephalus Gross motor delay Muscular hypotonia	Gross motor delay Speech delay Muscular hypotonia (for the first years of life)		Potential wheelchair dependency
Ear, nose, throat	Otitis media Hearing loss		Hearing loss	
Cardiorespiratory	Sleep apnoea (obstructive or central) Restrictive pulmonary disease (infancy) Hypertension Heart disease Increased mortality			
Dental		Tooth malocclusion		
Other	Sudden death		Obesity Chronic pain	

Initially, a 4-part engagement process was set up, comprising: a virtual meeting, for compilation of the topics and materials for discussion; an online review, for insight gathering using the Within3 platform (Within3 Inc., Lakewood, Ohio, United States); a virtual meeting, to present the findings from the online review and reach consensus on actions; and a final virtual meeting to reach agreement. During this review process, the Steering Committee drafted a list of variables collected in relation to achondroplasia and individually selected the 10 parameters they considered the most useful for studying the natural history of the condition and outcomes associated with treatment. They then reviewed the list of parameters and identified any items that had been omitted. The revised list was discussed by the committee during subsequent teleconference calls and virtual meetings and the final core dataset was determined. The project was funded by BioMarin Pharmaceutical (Novarto, CA).

## Results

### Achondroplasia information currently being collected across the EMEA region

According to the Steering Committee, a range of real-world information on achondroplasia is currently being collected at multiple, specialized clinical sites in the EMEA region, and is listed in Additional file 1: Table 2. Whereas many commonalities exist in the data collected (e.g. auxological measurements, medical history, surgical interventions, sleep studies, and genetic diagnosis), the exact data elements, the methods used to collect and store them (whether electronic or paper medical records), and the frequency of collection vary across sites.

### Selection of parameters for prospective data collection

The Steering Committee identified 65 items of importance in the study of achondroplasia. Most are specific to the condition, and several are collected as standard in routine clinical practice. The committee was then asked to each identify the items they considered the most relevant for a prospective registry of the natural history of achondroplasia and outcomes associated with treatment. Of the 65 original items, 23 were selected (Additional file 1: Figure), most commonly auxiological measures, sleep studies, quality of life, and neurological development. Of the 23 items, data on 11 are routinely collected by at least half of the HCP at their clinics (Additional file 1: Table 3).

Items considered most relevant by the AOR were similar to those selected by the HCP (Additional file 1: Figure), but included other factors including birth weight, mobility, device (positive ventilation and limb lengthening), and wheelchair use. Parameters considered important by both HCP and AOR included body length at birth, spinal deformities, pain, limb lengthening, and social and personal independence.

**Fig. 1:**
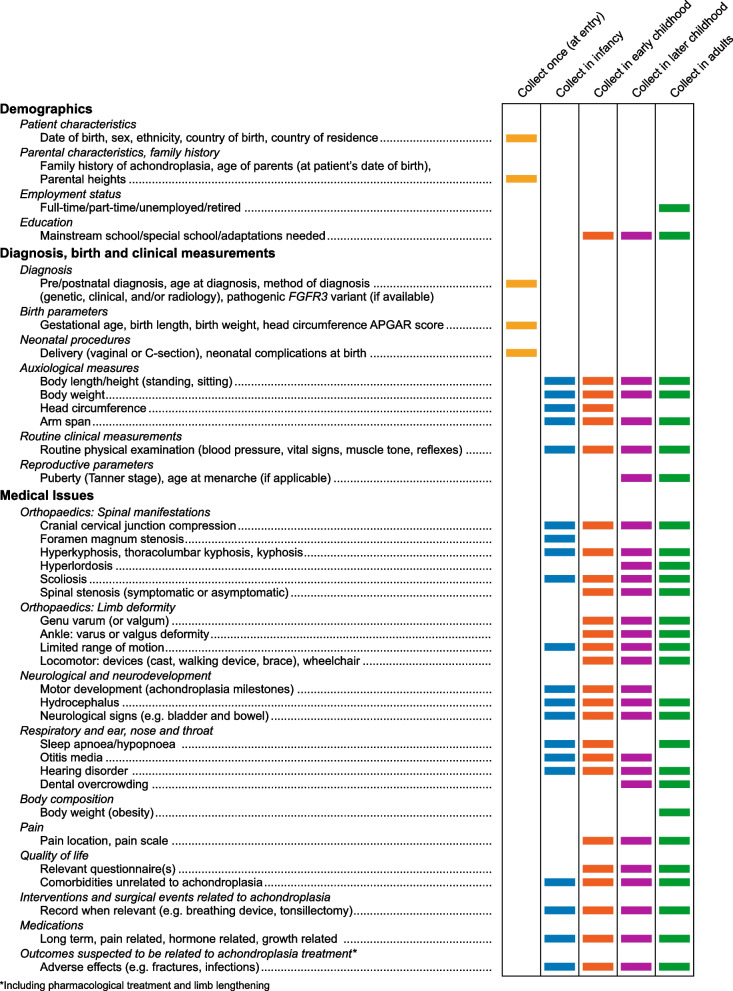
Data elements recommended for inclusion in prospective achondroplasia registries according to age group. *Note*: the decision to perform any investigation or document specific data is at the discretion of the treating physician, and some data elements are collected at baseline only

Data elements were reviewed during subsequent virtual meetings and teleconference calls, and consensus reached on the core set considered of greatest value for a standardized, international, prospective achondroplasia registry. The recommended data elements are shown in Fig. 1, grouped into three categories: demographics; diagnosis and patient measurements; and medical issues (including investigations and surgical events, medications, and outcomes possibly associated with achondroplasia treatments). Several of the elements only need to be recorded once (at entry into the study) or at specific times during the life course, whereas others would be collected at study entry and regularly thereafter, reflecting the natural history of the condition and the manifestations at each stage in the life course (Table 1) [1, 2, 7, 12, 16–20].

## Discussion

Our findings revealed both commonalities and notable differences in data collection for achondroplasia across EMEA. Of the 23 parameters given the highest priority by HCP, fewer than half are routinely measured, indicating disparity between which information is collected and what is considered essential; it may also be indicative of the practicalities and feasibility of data collection in the clinic. Our findings show some agreement between the priorities of AOR and HCP, but they also indicate differences in the value placed on the type of information collected.

Potential challenges also exist regarding the completeness of data. Wide variations exist across healthcare systems in terms of who (patient, primary care practitioner, or specialist), where (hospital database, patient records), and how (in specified ‘fields’ or as free text, in paper or electronic files) individual patient data are stored and who has access.

### Relevance of data according to age

One important consideration for collecting real-world data is the collection of pragmatic and relevant information on the natural history of the condition at the appropriate times during the affected person’s life course (i.e. according to age group). Onset of manifestations and complications in achondroplasia in different age groups is outlined in Table 1, which has informed the inclusion of parameters in Fig. 1 for collection of information across age groups.

In achondroplasia, impaired bone development in infants (birth to < 2 years) may lead to complications that can put them at risk for sudden death, giving the first years of life a greater sense of urgency and attentiveness. Up to four-fifths of infants with achondroplasia have sleep-disordered breathing during early infancy [21], and concomitant increased apnoeic events and decreased arousal response may be responsible for sudden unexpected deaths. Other complications during infancy include otitis media [7], hypotonia, and delayed motor developmental milestones [2].

After the age of 2 years, complications such as lower limb deformity, hearing loss, and speech impairments become more apparent. Children with achondroplasia also follow a specific pattern of motor developmental milestones despite age-appropriate cognitive development [22]. Impaired bone growth continues to affect body functions with the transition into childhood and early adolescence, potentially causing ENT, respiratory, and orthopaedic manifestations that can adversely affect developmental milestones such as speech and mobility if left untreated. Midface hypoplasia can contribute to sleep-disordered breathing [23] as well as hearing impairment and orthodontic issues, requiring specialist evaluation [24]. Hearing and speech impairment may persist during the teenage years [7], and the psychosocial and functional implications of living with achondroplasia may become more pronounced [8, 22, 25]. Other common problems during the teenage years include lack of weight control, reduced muscle strength, and lumbar hyperlordosis [24].

In adults with achondroplasia, complications can include spinal stenosis, chronic pain, obstructive sleep apnoea, hearing loss, and obesity, all of which may need specialist evaluation and treatment.

### Need for real-world evidence in achondroplasia, and the challenges

**Fig. 2:**
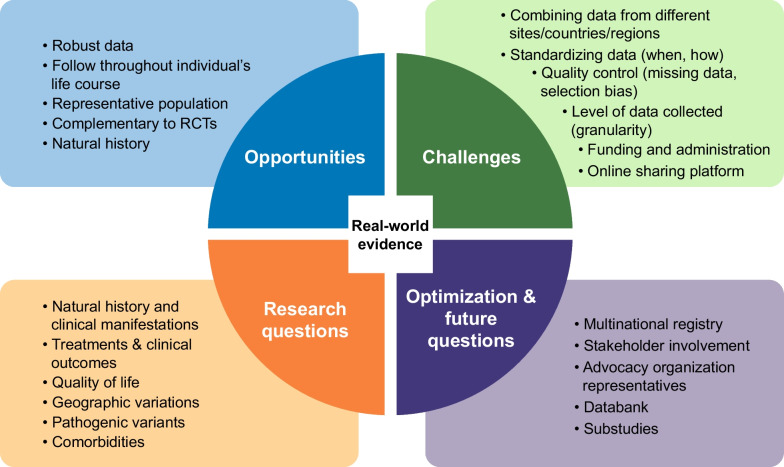
Role of real-world evidence in providing insights into the natural history of achondroplasia, management of achondroplasia, and clinical outcomes associated with treatment

The key benefits, challenges, research questions, and future directions of RWE are illustrated in Fig. 2. Randomized controlled trials (RCT) provide evidence of the safety and efficacy of new treatments but are subject to strict enrolment criteria that limit the representativeness of the population and are conducted over a relatively short period. In contrast, RWE is gathered from a variety of sources and is more representative of the population with a condition.

The challenges of setting up an international registry are substantial and multifactorial. Standardization of data, as well as implementing quality control measures, is imperative to ensure harmony, and will require substantial funding and administrative support. The legalities of data sharing, ethical issues, and patient confidentiality will also need to be addressed. For achondroplasia, in addition to being a rare condition, the situation is made more complex by the different pathways of patient care across—sometimes within—countries, and different specialities involved, resulting in lack of longitudinal data across age groups. However, the benefits in terms of answering research questions are enormous, providing insights into the challenges and natural history of the condition, geographic variations, comorbidities, psychological issues, and quality of life, as well as reporting clinical outcomes associated with treatment. Rare disease registries can also improve the quality and efficiency of RCTs by informing on sample size calculation; they have also been used as historical controls in single-arm clinical trials. In addition, post-regulatory approval registries provide long-term data on clinical outcomes, especially safety, from patients treated in routine clinical practice [26].

Pooling good-quality, long-term data from individuals across clinical practices and geographical boundaries would generate a more robust and representative picture of the medical and healthcare challenges faced by people with the condition and the outcomes associated with treatment. From the clinician’s perspective, the key aim of such a registry is to better understand the effect of achondroplasia throughout the life course, ultimately leading to improvements in management and support that are appropriate and relevant to the affected person throughout their life. The availability of RWE will also help HCP understand the effect of treatment approaches on social and educational outcomes. From the perspective of people with achondroplasia, the aims are to improve the knowledge of the condition, support scientific research, provide more evidence-based based information for families and patients, and identify factors influencing quality of life.

### Recommendations for documenting data from routine clinical practice

Whereas registry studies are generally less rigorous than RCTs, data quality remains vital. Before setting up a registry, stakeholders should first consider the key clinical questions that can be addressed by such an enterprise. These questions will help determine which data elements are essential to be collected. Limiting the number of elements to those considered of greatest value to both patients and HCP but are also feasible to collect and reflect the standard of care will likely increase the long-term success and optimize the data quality of such a project. Furthermore, the key list of data elements should serve as the starting position, with the flexibility and agility to capture additional data according to country-specific needs as well as to reflect changes in practice and availability of new treatments.

Standardizing the capture of data (e.g. auxological measurements) would improve the overall quality and could be achieved by using online tools (e.g. voluntary instructional videos for measuring arm span) and checklists. Standardizing data collection has the potential to allow pooling of datasets from different sites, regions, or countries. This could be achieved by developing a platform that can be accessed by HCP in different countries or a single access point for recording information on multidisciplinary care for an individual patient. While challenging, the development of a template embedded into patients’ electronic records, with drop-down lists of predefined items, would also facilitate the collection of data, ensure continuity of data collection, and facilitate progress reports.

Use of a condition-specific quality-of-life tool may help standardize data on pain, quality of life, and psychological issues, but is not generally included in the standard of care. Additionally, detecting tangible changes in quality of life takes time and necessitates comparison groups or historical matched controls. Capture of such information would need to be done on a voluntary basis and should be limited in extent to minimize the burden for patients and clinicians. Where possible, empowering patients or carers to access their data online and provide inputs may help significantly improve collection of such information. Other questionnaires could focus on daily independent living questions, self-esteem, and parent or carer perception of the child’s progress. Currently, several different quality-of-life tools have been used with varying success in clinical trials and natural history studies to measure quality of life [27–29]. There is a need and opportunity also to ascertain the most useful validated tool for prospective collection of quality-of-life measures in achondroplasia.

### Advocacy organization perspectives

From the AOR perspective, a well-conducted registry can improve understanding of the natural history of achondroplasia as well as support research and development, in turn providing more evidence-based information for people with achondroplasia and their families, and identifying factors influencing long-term quality of life such as independence, personal relationships, health, and wellbeing.

Advocacy groups often obtain valuable and comprehensive data from their members through surveys and social media, but most of this information is not standardized and may not even be analysed, losing a valuable opportunity to improve understanding within the community. Advocacy groups face sizeable obstacles when attempting to set up a registry, including funding, proprietary information, and resources (e.g. data collection and third-party governance). The development of a common international registry that adheres to the principles of ‘findability, accessibility, interoperability, and reuse of digital assets’, set up in close collaboration between healthcare professionals and advocacy groups, would ensure that the data collected are relevant and meet the requirements of all stakeholders. Two such ventures are the European Registry for Rare Bone and Mineral Conditions (EuRR-Bone; https://eurr-bone.com/) and CrescNet (https://www.crescnet.org/), where a standardized dataset aligned with the elements presented in this paper has been incorporated into an achondroplasia-specific module.

Knowledge gained through data analysis from such resources would be continuing and evolving, and can be used by healthcare providers to improve patient care. The transformation of real-world data into useful information that improves the care of each individual and educates the broader community is a much needed and expected demand.

### Strengths and limitations

Opinions are those of the EMEA Steering Committee. This consensus offers a starting point for developing a robust, prospective, international registry on which to conduct research into achondroplasia.

## Conclusions

Achondroplasia can be associated with significant consequences, not only for patients and their caregivers, but also for society and healthcare systems. Thus, early identification and appropriate treatment of serious complications are crucial to improve clinical status and achieve better quality of life. Long-term, high-quality RWE is needed to provide insights into the natural history of achondroplasia, leading to better understanding of patient experiences and perspectives, as well as clinical outcomes associated with current and future therapeutic approaches. Establishing regional, nationwide, or multinational registries that collect a predefined set of data elements across age spans will provide real-time prospective and longitudinal information and may be valuable in answering clinical questions. Where participation in global or multinational registries is not feasible, collection of standardized data in a harmonized way would help in pooled analysis and potentially comparable and comprehensive evidence generation. Despite differences across regions, it should be feasible to document a minimum dataset at a country level, using the key elements identified during this process, with the flexibility to add additional country-specific criteria and to pool data to examine clinical outcomes associated with achondroplasia and its treatment.

## Supplementary Information

**Additional file 1: Appendix Table 1**. EMEA Achondroplasia Steering Committee.

## Data Availability

Not applicable.
