# Supplementary material for: Real-world evidence in achondroplasia: considerations for a standardized data set
Source: Orphanet J Rare Dis. 2023 Jun 26;18:166. doi: 10.1186/s13023-023-02755-w (PMC10294372; doi:10.1186/s13023-023-02755-w)
Supplement: Supplementary file 1 — Additional file 1: Appendix Table 1. EMEA Achondroplasia Steering Committee. [file 13023_2023_2755_MOESM1_ESM.docx]

**SUPPLEMENTARY APPENDIX**

**Contents**

[Appendix Figure. Data elements considered of greatest value for an achondroplasia registry by EMEA Steering Committee (note: only one advocate representative was present) 2](#_Toc105576654)

[Appendix Table 1. EMEA Achondroplasia Steering Committee 3](#_Toc105576655)

[Appendix Table 2. Data currently collected on achondroplasia according to the EMEA Achondroplasia Steering Committee (11 unique centres*). 5](#_Toc105576656)

[Appendix Table 3. Data* currently collected by HCP in the Steering Committee. 7](#_Toc105576657)

# Appendix Figure. Data elements considered of greatest value for an achondroplasia registry by EMEA Steering Committee (note: only one advocate representative was present)

ACH, achondroplasia; BMI, body mass index; ENT, ear, nose, throat; FM, foramen magnum; MRI, magnetic resonance imaging.

# Appendix Table 1. EMEA Achondroplasia Steering Committee

| **Member** | **Location/Organization** | **Clinic type, population served (e.g. urban), etc.** |
| --- | --- | --- |
| ***Healthcare practitioners*** |  |  |
| Melita Irving, Co-Chair | Guy's and St. Thomas' NHS Foundation Trust, Evelina Children's Hospital, London, UK | University hospital, tertiary care |
| Klaus Mohnike, Co-Chair | Department of Pediatrics, Otto-von-Guericke-University, Magdeburg, Germany | University hospital, tertiary care |
| Yasemin Alanay | Pediatric Genetics, Department of Pediatrics, School of Medicine, Acibadem Mehmet Ali Aydinlar University, Istanbul, Turkey | Affiliated university hospital, tertiary care |
| Moeenaldeen AlSayed | King Faisal Specialist Hospital & Research Center, Riyadh, Saudi Arabia | Tertiary care hospital.  Specialized genetic/metabolic clinic (25 million) |
| Genevieve Baujat | Hôpital Necker Enfants Malades AP-HP, France | University hospital, tertiary care |
| Tawfeq Ben-Omran | Genetic and Genomic Medicine Division, Sidra Medicine and Hamad Medical Corporation, Doha-Qatar | University hospital, tertiary care |
| Sandra Breyer | UKE Hamburg-Eppendorf, Department of Paediatrics, Hamburg, Germany | University Hospital - tertiary care  ICLD, Martin Zeitz Center for Rare Diseases |
| Valerie Cormier-Daire | Reference center for skeletal dysplasia, Paris University, Imagine institute, Necker Enfants Malades Hospital, Paris, France | University hospital, tertiary care |
| Pernille Axél Gregersen | Department of Clinical Genetics and Centre for Rare Diseases, Aarhus University Hospital, Aarhus, Denmark | Skeletal Dysplasia Clinic at University Hospital, tertiary care  ERNBOND Center |
| Encarna Guillén-Navarro | Medical Genetics Section, Department of Paediatrics. Virgen de la Arrixaca University Clinical Hospital; IMIB-Arrixaca; Faculty of Medicine, University of Murcia (UMU), Murcia, Spain | Skeletal Dysplasia Clinic in Tertiary Academic Hospital, Regional and National referral centre; ERNBOND expert Centre |
| Wolfgang Högler | Department of Paediatrics and Adolescent Medicine, Johannes Kepler University Linz, Linz, Austria | University hospital, tertiary care |
| Mohamad Maghnie | Department of Paediatrics, IRCCS Istituto Giannna Gaslini, and Department of Neuroscience, Rehabilitation, Ophthalmology Genetics, Maternal and Child-Health, University of Genova, Genova, Italy | Tertiary academic and national referral centre |
| Ola Nilsson | Division of Pediatric Endocrinology, Astrid Lindgren Children’s Hospital, Karolinska University Hospital, Stockholm, Sweden | Skeletal dysplasia clinic at University hospital/tertiary center, Regional and National referral centre; ERNBOND expert center |
| Angelo Selicorni | Pediatric Unit ASST Lariana, Como Italy | Member of the network of centres of reference for rare diseases for achondroplasia at regional level |
| Oliver Semler | University of Cologne, Faculty of Medicine and University Hospital Cologne, Department of Pediatrics, Cologne, Germany | University hospital, tertiary care |
| Sabine Sigaudy | Medical Genetics Department  Competence Center for skeletal dysplasia Hôpital Timone Enfant Marseille France | University hospital, tertiary care |
| Dmitry Popkov | National Ilizarov Research Center for Traumatology and Orthopaedics, Kurgan, Russia | Tertiary national referral centre |
| ***Advocacy organization representatives*** | | |
| Marco Sessa | President, Associazione per I’Informazione e lo Studio dell’Acondroplasia (AISAC), Italy | |
| Susana Noval | Fundación ALPE Acondroplasia, Spain | |
| Inês Alves | ANDO Portugal, Evora, Portugal | |

EMEA, Europe, Middle East, and Africa.

# Appendix Table 2. Data currently collected on achondroplasia according to the EMEA Achondroplasia Steering Committee (11 unique centres*).

| **Parameter** | **11 centres** |
| --- | --- |
| Auxologic/growth measurements (e.g. head circumference, weight, BMI) | 11 |
| Medical history | 8 |
| Surgeries procedures (neuro/orthopaedic/ENT) | 8 |
| Polysomnography/sleep disordered breathing/sleep disorders/sleep questions | 8 |
| Genetic diagnosis | 7 |
| Physical examination (with functionality)/physical function | 6 |
| MRI/cranial imaging/spine imaging/neuroimaging | 6 |
| Psychomotor development (motor milestones, fine motor skills, social) | 5 |
| Education information | 5 |
| Hearing problems/test | 4 |
| Locomotor exam/Joint ROM/deformities | 4 |
| Physical activities (including sports) | 4 |
| Prenatal history | 3 |
| Neonatal history | 3 |
| Pain | 3 |
| X-rays including bone age | 3 |
| Birth parameters/measurements | 3 |
| Laboratory tests | 2 |
| Walking test/mobility | 2 |
| Psychological problems/psychological issues | 2 |
| Therapy input/therapies | 2 |
| Therapy input/therapies | 2 |
| Neurological issues/bladder function/neurological exam | 2 |
| Ear/nose | 2 |
| Medications | 1 |
| Diet and food consumption | 1 |
| Age and height of parents | 1 |
| Teeth | 1 |
| Denver development (all four domains) | 1 |
| Home interaction/daily activities | 1 |
| Administrative data (e.g. name, date of birth) | 1 |
| Type of medical activity (e.g. consultation, hospitalisation), date | 1 |
| Surveillance test results | 1 |
| Interventions | 1 |
| Motor function (gross motor function measure test) | 1 |
| Use of aids | 1 |
| Aims of patient and family | 1 |
| Thorax asymmetry and management | 1 |
| Neurological examination | 1 |
| Fatigue questions | 1 |
| Referring physician name | 1 |
| Familial case/not familial case | 1 |
| Natural history | 1 |

*Responses from 11 Steering Committee members from France (n=3), Germany (n=2), Italy (n=2), Saudi Arabia (n=1), Sweden (n=1), Turkey (n=1), UK (n=1). Free-text responses were provided by the available members of the Steering Committee. Where possible, responses have been grouped.

EMEA, Europe, Middle East, and Africa.

# Appendix Table 3. Data* currently collected by HCP in the Steering Committee.

| **Data collected by ≥50% of HCP** | **Data collected by <50% of HCP** |
| --- | --- |
| Sleep studies/polysomnography | Foramen magnum stenosis |
| Arm span | Quality of life |
| Seated height | Neurological impairments |
| Motor development | Mobility aid use |
| MRI (brain and spine) | Spinal deformities/scoliosis |
| Head circumference | Pain (joint/knee/back) |
| Mutation (de novo/inherited) | Birth length |
| Limb lengthening | Age of milestone achievement |
| Assessment: Polysomnography | Social autonomy |
| Family history of ACH | X-rays |
| Ear, nose and throat | Personal autonomy (hygiene) |
|  | Physiotherapy |

*From the 23 items identified by the Steering Committee as important for a registry on achondroplasia.
